# Second primary malignancies after treatment for malignant lymphoma

**DOI:** 10.1038/sj.bjc.6602731

**Published:** 2005-08-16

**Authors:** A Okines, C S Thomson, C R Radstone, J M Horsman, B W Hancock

**Affiliations:** 1 YCR Academic Unit of Clinical Oncology, Weston Park Hospital, Whitham Road, Sheffield S10 2SJ, UK; 2 Trent Cancer Registry, 5 Old Fulwood Road, Sheffield S10 3TG, UK

**Keywords:** second primary cancer, malignant lymphoma, matched case–control study

## Abstract

To determine the incidence and possible causes of second primary malignancies after treatment for Hodgkin's and Non-Hodgkin's lymphoma (HL and NHL). A cohort of 3764 consecutive patients diagnosed with HL or NHL between January 1970 and July 2001 was identified using the Sheffield Lymphoma Group database. A search was undertaken for all patients diagnosed with a subsequent primary malignancy. Two matched controls were identified for each case. Odds ratios were calculated to detect and quantify any risk factors in the cases compared to their matched controls. Mean follow-up for the cohort was 5.2 years. A total of 68 patients who developed second cancers at least 6 months after their primary diagnosis were identified, giving a crude incidence of 1.89% overall: 3.21% among the patients treated for HL, 1.32% in those treated for NHL. Most common were bronchial, breast, colorectal and haematological malignancies. High stage at diagnosis almost reached statistical significance in the analysis of just the NHL patients (odds ratio=3.48; *P*=0.068) after adjustment for other factors. Treatment modality was not statistically significant in any analysis. High stage at diagnosis of NHL may be a risk factor for developing a second primary cancer.

‘Late effects’ of lymphoma treatment are being increasingly documented as more patients are surviving their disease. Arguably the most serious of these late effects is the development of a second primary malignancy (SPM).

An association between HL and other tumours was first documented in 1957 (Moertal and Hagendorn, 1957) when a combined review and case series of 120 patients was published. Since then, numerous studies have described a significantly increased risk of second cancers in these patients. Initially, the major risk was thought to be from second acute leukaemias (Canellos *et al*, 1975; Pederson-Bjergaard and Larsen, 1982; Valagussa *et al*, 1986) and associations were quickly demonstrated with certain chemotherapy regimens such as MOPP (mustine, Oncovin, procarbazine, prednisone) (Valagussa *et al*, 1986; Kaldor *et al*, 1990; Tura *et al*, 1993). Splenectomy has also been proposed as a risk factor (Pederson-Bjergaard and Larsen, 1982). The risk of developing acute leukaemia appears to peak approximately 5 years after onset of chemotherapy (Tura *et al*, 1993) and plateau by approximately 10 years (Tucker *et al*, 1988). In contrast, the risk of second solid cancers appears to increase with time from diagnosis (Valagussa *et al*, 1986; Tucker *et al*, 1988; Swerdlow *et al*, 1992), which means that they, in fact, are the major risk to long-term survivors of HL. Much controversy exists as to the major risk factors for developing second solid cancers, with many investigators finding an increased risk in all treatment groups (Tucker *et al*, 1988; Boivin *et al*, 1995; Dores *et al*, 2002). However, that there is an increased risk with younger age (Swerdlow *et al*, 2000) and particularly of breast cancer in women treated with radiotherapy (RT) is now widely accepted (Janjan *et al*, 1988; Hancock *et al*, 1993; van Leeuwen *et al*, 1994a; Aisenberg *et al*, 1997).

Noticeably, less literature exists concerning second cancers in patients treated for NHL. This may, in part, be a reflection of the higher average age at diagnosis, the poorer survival and hence the reduced availability of patients for long-term follow-up. However, a significant risk of myelodysplasia and acute leukaemia has been demonstrated in patients treated with a variety of treatment regimens such as radiotherapy alone and high-dose chemotherapy with autologous stem cell support (Stone, 1994; Micallef *et al*, 2000). An excess risk of developing certain solid cancers have also been described, particularly bladder, kidney, lung, malignant melanoma, HL (Travis *et al*, 1991) and brain tumours (Travis *et al*, 1993). Of these, a positive association was demonstrated between chemotherapy and bladder cancer (Travis *et al*, 1993), and between radiotherapy and lung, bladder and bone malignancies (Travis *et al*, 1991). However, other studies have failed to demonstrate any excess risk of these tumours in any treatment groups. A study published in 1990 (Lavey *et al*, 1990) described an incidence of second solid tumours similar to that expected in the general population, with significant differences only present for salivary gland tumours (both overall and in patients treated with chemotherapy alone) and malignant melanomas (overall).

Where an elevated risk of solid cancers has been demonstrated, it appears to remain significantly increased up to 20 years after lymphoma diagnosis (Travis *et al*, 1993).

While it is important to determine the incidence of second primary malignancies in survivors of both HL and NHL, it is more vital to define the risk factors for each type of malignancy, so that either these can be modified or patients at particular risk can be closely monitored.

## PATIENTS AND METHODS

The study was a nested case–control design. In total, 3764 consecutive patients diagnosed with HL or NHL between the January 1970 and 28 July 2001 were identified using the Sheffield Lymphoma Group database. For all patients, information on sex, date of birth, age at diagnosis, date of diagnosis, histology, stage at diagnosis, presenting site, date last seen, vital status, duration of follow-up and occurrence of second cancers was extracted from the database. Patients who were only seen once at Weston Park Hospital and completed all of their treatment elsewhere were immediately excluded from the study. For the purpose of this study, SPM is defined as any site of second malignancy either *in situ* or invasive, except Kaposi's Sarcoma in HIV positive individuals, diagnosed at least six complete months after lymphoma diagnosis but before death. Any cases of SPM diagnosed in patients who received initial treatment for their lymphoma at other centres were excluded, as were all malignancies diagnosed within 6 months of lymphoma diagnosis and also cases of third primary malignancies.

Two controls were identified for every case, matched as closely as possible for their primary diagnosis (HL or NHL), sex, age at diagnosis (±2 years), duration of follow up (±1 year of time from primary diagnosis to the second cancer diagnosis), and year of diagnosis (±10 years). The group of second cancer cases and their matched controls is the main study sample. For both the cases and their controls, additional information was extracted from their notes on the type and dates of treatment received, including chemotherapy regimens, radiotherapy doses and fields, splenectomy, stem cell support, and also date and diagnosis of the subsequent primary malignancy. Information was also sought as to the smoking status of the patients, but this was often (>35%) incomplete.

Matched conditional logistic regression (Breslow and Day, 1980) was used to determine whether any of the variables were risk factors for the development of an SPM. Three separate groups of analyses were undertaken, one on all patients (either HL or NHL disease as the first primary malignancy), one based only on NHL patients and finally one based only on the HL patients. Since matching was performed on age, sex, whether HD or NHL disease as the first primary disease and the duration of follow-up, none of these variables, or those derived from them, were included in the analyses. However, because matching on time of diagnosis was only performed within 10 years, in these analyses, adjustment for the effect of time of diagnosis was performed by including it as a covariate in the model. Thus, the variables decade of primary diagnosis (1970–1979, 1980–1989, 1990–2001), presenting site (nodular disease, extra-nodular disease, other (B symptoms (4), cord compression (1), superior vena cava obstruction (1) and alcohol pain (1)), stage (low: I and II; high: III and IV) and treatment (radiotherapy only, chemotherapy only, radiotherapy and chemotherapy, surgery alone/unknown treatment/no treatment) were included in all three analyses. Additionally, for the NHL patients, histology type for NHL (high grade: high-grade B cell or high-grade T cell; other: low-grade B cell, low-grade T cell or unknown grade) was also analysed. For the analysis of the HL patients, histology type for HL (nodular-sclerosing; mixed cell; other: lymphocyte depleted, lymphocyte predominant or unknown) and having a splenectomy or not were included. The groups for classifying histology for both HL and NHL were necessary due to the small numbers in some of the groups. Odds ratios (OR), 95% confidence intervals (CI) and *P*-values were estimated when the variables were examined separately in univariate models and all together in multivariate models. Odds ratios were not presented when there were fewer than 5% of either cases or controls in the risk factor level. All analyses were performed in Stata version 7.0 (StataCorp, 2001).

## RESULTS

**Figure 1:**
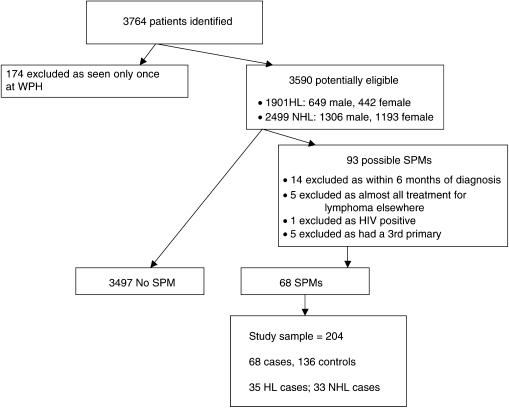
Total population, exclusions and distribution of cases and controls within the study sample.

In total, 3764 consecutive patients diagnosed with HL or NHL between the January 1970 and 28 July 2001 were identified using the Sheffield Lymphoma Group database. For the whole population, the median duration of follow-up is 2.79 years, mean 5.19 years. There were 174 patients who were only seen once at Weston Park Hospital and completed all of their treatment elsewhere who were immediately excluded from the study. Figure 1 gives the details of the remaining 3590 patients, as well as shows why 25 of the 93 cases of second malignancies identified were excluded from the analysis. Therefore, 68 cases of SPM met the inclusion criteria, giving a crude incidence of 1.89% overall: 3.21% among the patients treated for HL, 1.32% in those treated for NHL. Most common were bronchial, breast, colorectal and haematological malignancies. All SPMs included in the study are shown in Table 1. Thus, matched conditional logistic regression was performed on 68 cases (35 HD, 33 NHL) and 136 controls to determine whether any of the variables were risk factors for the development of an SPM.

### All patients

When the matched conditional logistic regression was performed on the 68 cases and 136 controls together, only decade of diagnosis (*P*=0.029) was significant in both the univariate and multivariate models (Table 2). Patients diagnosed in 1980–1989 had half the risk of developing an SPM than patients diagnosed in 1970–1979. Similarly, patients diagnosed in 1990–2001 had less than a tenth of the risk of developing an SPM than patients diagnosed in 1970–1979 in the multivariate model. Patients diagnosed with either stage III or IV disease had a higher risk of developing an SPM (1.7 times) than those diagnosed with stage I or II disease, and those patients given chemotherapy, either alone or in combination had lower risks of developing an SPM than those patients just having radiotherapy, although none of these were statistically significant.

### NHL patients

The analysis of the NHL patients only showed that patients with high stage disease had 3.5 times the risk of developing an SPM than those diagnosed with low stage disease although this was marginally nonsignificant after adjustment for other factors in the multivariate model (*P*=0.068). Patients with NHL that was not high grade had a lower but nonsignificant risk of developing SPM than those with high grade NHL, and patients given chemotherapy alone or in combination generally had a lower risk than those given only radiotherapy, but these were not significant (Table 3).

### Hodgkin's lymphoma patients only

Table 4 presents the univariate and multivariate results for the matched conditional logistic regression for the 35 and 70 HL cases and controls, respectively. None of the variables reached statistical significance for predicting whether the patient would develop an SPM for those patients diagnosed with HL, in either the univariate or multivariate models.

## DISCUSSION

There can be no doubt that the long-term survivors of malignant lymphoma are at increased risk of developing second cancers and that this risk results mainly from the oncogenic sequelae of cytotoxic and/or radiation therapy (Connors, 2003; Hancock, 2003). It was surprising that we could not replicate these results, although due to the low numbers (four cases, seven controls) in the minimal treatment (no chemotherapy and/or radiotherapy) group the reference category for the analyses of treatment was generally taken as those patients having radiotherapy alone. It is quite difficult to assess the actual risks (Henry-Amar, 2000) but is likely that incidence of leukaemia in the first 10 years after chemotherapy for HL is in the order of 5–10% (with a relative risk exceeding 10, compared with a matched reference population). Similar relative risks are seen for secondary NHL 5–15 years post-treatment. However, these conditions have a low natural incidence so the actual number of cases is small. More seriously, for both HL and NHL, the incidence of second solid tumours approaches 20% after two decades (with a relative risk of only between 1.5 and 2.5, but accounting for larger number of cases). Many of the earlier reports were from specialist centres or clinical trial collaboratives leading inevitably to patient selection. Our study deals with a relatively nonselected series of consecutive patients seen at a provincial lymphoma centre.

### All lymphomas

Of the 68 SPMs identified in this study, bronchial and breast carcinomas were most frequent, and solid cancers overall were much more common than haematological malignancies. This is supported by similar findings in a 1990 study of second malignancy risk after lymphoma treatment (Moertal and Hagendorn, 1957).

Conditional logistic regression analysis showed a nonstatistically significant increase in the risk of developing an SPM for those with higher stage tumours. The observed increase in the risk of developing an SPM for those diagnosed in the decade 1970–1979 compared with those diagnosed in 1980–1989 and 1990–2001 is almost certainly an artefact of the design with matching having been performed on length of follow-up. The patients first diagnosed in 1970–1979 had a longer follow-up time in which to develop a second primary compared to the later decades, and are likely to have had more controls diagnosed in the later periods. This is therefore of no particular interest.

### Non-Hodgkin's lymphoma

The incidence of SPMs in this study population is less than has been reported in previous studies and this is especially marked in the NHL patients, in whom a crude incidence of 1.32% is reported here, compared to the 4.22 (Travis *et al*, 1991) to 8.77% (Travis *et al*, 1993) reported in the literature in both small studies (Lavey *et al*, 1990) and large multicentre analyses (Travis *et al*, 1991). This may be a reflection of the short duration of follow-up overall for both HL and NHL patients in this study (mean 5.19 years, median 2.79 years) compared to a median of 5.5 years (Lavey *et al*, 1990), or mean of 7.4 years (Travis *et al*, 1993) in other studies, but the possibility that not all cases of SPM have been recorded on the database cannot be excluded. A simple way of increasing the mean duration of follow-up and the overall incidence would be to limit the cohort to patients who survived longer than 6 months. This would give a more accurate representation of the incidence, as patients who have died within 6 months have not actually had the chance to develop an SPM, by this study's definition. Additionally, the initial determination of those developing an SPM was performed only 6 months after the cutoff for inclusion in the whole cohort thus reducing the mean follow-up time. Finally, 145 of the 3764 patients were lost to follow-up, and so may have in fact developed an SPM that is not known about. These patients comprised those who were followed-up at other hospitals, those who went on to receive some of their treatment at local district general hospitals and those who were discharged from clinic but whose General Practitioners did not return the two-yearly postal follow-up requests.

There was only a single case of leukaemia that occurred in this patient group, which is probably significantly lower than the 11 out of 517 patients with leukaemia or myelodysplasia reported in a 1983 study (Greene *et al*, 1983), or the four out of 61 patients reported in 1996 (Travis *et al*, 1996), although we would need the duration of follow-up for each of the studies to be comparable to be certain of this. However, in the former study, the high incidence is probably due to the intensity of the treatment received by the patients; seven out of the nine patients had had radical radiotherapy and one had six cycles of MOPP chemotherapy, despite eight of the cases having had an ‘indolent NHL subtype’. Equally, in the latter study, all patients had received low-dose total body irradiation and chemotherapy, in contrast to our study population, who had received treatment as diverse as node excision only and through to intense combined modality regimens, thus making comparison difficult.

Solid tumours were far more common in our patients, with the most frequently observed SPM being bronchial carcinoma. This supports the findings of a 1991 multi-centre study (Travis *et al*, 1991), which reported 274 cases of lung cancer (more than any other site) in 29 153 patients treated for NHL. The same study found colorectal carcinomas to be the next most frequent (186 cases), followed by breast and prostate cancers. Skin cancers had been excluded from the analysis. In the current study, breast carcinoma and skin cancers were common, but only two cases of prostate cancer were recorded. Similar findings were also published in 1993 (Travis *et al*, 1993), where the investigators reported bronchial carcinoma as the most common SPM, followed by colorectal, breast and prostate carcinomas.

From the matched conditional logistic regression analysis, the one variable that almost reached statistical significance was stage at diagnosis, which was only marginally nonsignificant (*P*=0.068), with patients of stages III and IV at higher risk of developing an SPM compared to those presenting with either stage I or II disease. This result could prove to be an independent risk factor in a larger study, but may also have been confounded by the fact that stage I and II disease is most likely to be treated with local radiotherapy, whereas stage III and IV disease is more likely to have been treated with combination chemotherapy for high-grade disease or prolonged chlorambucil±radiotherapy for low-grade disease. Previous studies have shown positive correlates between radiotherapy dose and leukaemia SPM (Greene *et al*, 1983), radiotherapy and leukaemia, lung, bladder and bone SPMs, and chemotherapy and leukaemia and bladder SPM (Travis *et al*, 1991). As the number of patients in this study was small, separate analyses were only carried out for the two most common SPMs, lung and breast, for all lymphomas together, so no comparison to other results can be made. Histological subtype did not reach statistical significance in this study (as a risk factor for developing an SPM). A recent study of patients with mantle cell lymphoma (Barista *et al*, 2002) reported a crude incidence of 7.05% of patients developing an SPM. Clearly, this is much higher than reported here, but lies within the range reported in the literature, so this sort of lymphoma may not prove to be an independent risk factor.

### Hodgkin's lymphoma

The incidence of SPMs in this group (3.21%) is slightly lower than most previously reported (Valagussa *et al*, 1986; Prior and Pope, 1988; Tucker *et al*, 1988; Cimino *et al*, 1991; Swerdlow *et al*, 1992, 1997; Foss Abrahamsen *et al*, 1993), but lies within the overall range of 2.17 (Prior and Pope, 1988) to 7.53% (Swerdlow *et al*, 1997).

Here, six cases of haematological malignancy were recorded (0.55%), which is noticeably lower than the 4.35% (Pederson-Bjergaard and Larsen, 1982) or 4.37% (Tura *et al*, 1993) reported elsewhere. However, both studies selected patients treated with a combination of MOPP chemotherapy and radiotherapy. More recently, a study in a more heterogeneous population reported an incidence of 2.27% (van Leeuwen *et al*, 1994b), which although clearly still much higher than reported here, is more comparable to this study. As stated previously, it is possible that the reduced incidence reported here is a result of the relatively short duration of follow-up and potentially incomplete records.

Solid cancers were more common than haematological SPMs, as is the case in the literature (Canellos *et al*, 1975; Valagussa *et al*, 1986; Prior and Pope, 1988; Tucker *et al*, 1988; Cimino *et al*, 1991; Foss Abrahamsen *et al*, 1993; van Leeuwen *et al*, 1994a; Boivin *et al*, 1995; Swerdlow *et al*, 1997). Here, breast carcinoma was the most common solid SPM, with seven cases (1.66%) recorded. Again, this figure is much lower than the 2.82% (Hancock *et al*, 1993) or 12.61% (Aisenberg *et al*, 1997) previously reported. However, in the former study, the mean duration of follow-up was 5.19 years, and in the latter, median 18 years and all patients were aged less than 60 and had stage 1 or 2 disease and so this was also a highly selected patient group. Bronchial carcinoma occurred in six patients, an increased risk has also been previously reported (Kaldor *et al*, 1990; van Leeuwen *et al*, 1995; Swerdlow *et al*, 2001). In the first study of 98 cases and matched controls (Kaldor *et al*, 1990), the investigators found a higher risk following CT than RT. The second study (van Leeuwen *et al*, 1995) related radiation dose received by the affected area of lung to risk in their 30 cases and controls. Most recently, in a study of 88 cases out of a cohort of 5519 (Swerdlow *et al*, 2001), borderline significance was found for MOPP chemotherapy compared to other treatment regimens. In the current study, all six of the cases received mantle RT, and two received CT (either MOPP or L[Leukeran]OPP) for relapse.

None of the variables were statistically significant in the conditional logistic regression analyses, although patients who had a splenectomy had a nonsignificantly higher risk of developing an SPM (*P*=0.47) in the multivariate model. It is possible that splenectomy would have been statistically significant if we were able to restrict the analysis to leukaemias, as an increased risk has been reported of 21 out of 557 splenectomised patients compared to one out of 145 not splenectomised (*P*=0.01) (Tura *et al*, 1993) but the numbers in our study are too small for such an analysis.

### Bronchial carcinoma SPM

When additional logistic regression analysis was undertaken on the 13 triples for the patients having an SPM in the lung, there were no statistically significant risk factors, although treatment modality bordered on significance when it was grouped up as the rest *vs* combination RT and CT (odds ratio: 3.70; 95% CI: 0.72, 18.97; *P*=0.12). Treatment modality might well be statistically significant if the sample size allowed valid division into HL and NHL patients, or the treatment modalities did not need to be grouped up. Different treatment modalities have been reported to affect the risk of bronchial carcinoma in patients with HL; although two studies concluded that chemotherapy was the most important risk factor (Kaldor *et al*, 1990; Swerdlow *et al*, 2001) the third showed radiation dose to be statistically significant (van Leeuwen *et al*, 1995).

### Breast carcinoma SPM

Further logistic regression analysis was carried out on the 11 triples for the cases having an SPM in the breast. Since one of the major observations previously reported for HL cases is that there is an increased risk with breast cancer in women treated with radiotherapy (RT) (Janjan *et al*, 1988; Hancock *et al*, 1993; van Leeuwen *et al*, 1994a; Aisenberg *et al*, 1997; Swerdlow *et al*, 1997), we examined whether having any radiotherapy (either alone or in combination) affected whether or not there was a breast SPM, for all patients. We found that all of the cases (i.e. those 11 having a breast SPM, seven of which were HL patients) were given some form of RT, while non of the patients who were not given RT, either alone or in combination, went on to develop a breast SPM. Thus, while it was not possible to test this statistically, this finding does appear to support the view that giving RT to HL patients does increase the risk of developing breast cancer.

## CONCLUSION

In this study of all patients treated in one provincial Centre, having high stage disease at presentation, especially for NHL patients, was only marginally nonsignificant. This may be a significant risk factor in a larger study. Other individual factors may be important for different SPMs and only with larger numbers of patients and longer follow-up can these be elucidated.

## Figures and Tables

**Table 1:** Distribution of SPMs by lymphoma diagnosis and sex

**Type of SPM**	**HL male**	**HL female**	**NHL male**	**NHL female**	**Total**
Bronchial carcinoma	4	2	5	2	13
Breast carcinoma	0	7	0	4	11
Colorectal carcinoma	1	0	4	3	8
Skin cancers	4	0	3	1	8
Leukaemia or myelodysplasia	4	2	1	0	7
Stomach carcinoma	0	1	3	0	4
Multiple myeloma	1	0	0	1	2
Prostate carcinoma	0	0	2	0	2
Brain tumours	1	0	1	0	2
Pancreatic carcinoma	1	0	1	0	2
Metastatic carcinoma, unknown primary	1	0	1	0	2
NHL	1	1	0	0	2
Bladder carcinoma	1	0	0	0	1
Cholangiocarcinoma	1	0	0	0	1
Ovarian carcinoma	0	1	0	0	1
Cardiac sarcoma	0	1	0	0	1
Anal carcinoma	0	0	0	1	1
					
Total	20	15	21	12	68

SPM=second primary malignancy.

**Table 2:** Odds ratios (OR), 95% confidence intervals (CI) and numbers of exposed cases (ca) and controls (co) for risk factors for both HL and NHL patients together

**Factor**	**Category^a^**	**All patients univariate OR (95% CI) ca 68, co 136**	**All patients multivariate OR (95% CI)**
Decade of diagnosis (years)	1970–1979	1.00	1.00
		30, 50	
	1980–1989	0.50 (0.18–1.39)	0.55 (0.19–1.60)
		28, 57	
	1990–1999	0.08^b^ (0.01–0.75)	0.09^c^ (0.01–0.94)
		10, 29	
			
Stage at diagnosis	Low	1.00	1.00
		43, 91	
	High	1.29 (0.72–2.32)	1.69 (0.72–3.95)
		25, 40	
			
Presenting site at diagnosis	Nodal disease	1.00	1.00
		54, 94	
	Extra-nodal disease	0.56 (0.25–1.27)	0.69 (0.26–1.85)
		13, 36	
	Other	—	—
		1, 6	
			
Treatment given	RT only	1.00	1.00
		31, 54	
	CT only	0.87 (0.43–1.75)	0.63 (0.23–1.68)
		19, 38	
	RT and CT	0.62 (0.28–1.39)	0.63 (0.26–1.50)
		14, 37	
	Surgery only/no treatment/treatment u/k	0.99 (0.26–3.84)	1.05 (0.21–5.15)
		4, 7	

a Reference category stated first.

b *P*=0.029.

c *P*=0.044.

—, Odds ratio not presented as fewer than 5% of either cases or controls in the risk factor level.

**Table 3:** Odds ratios (OR), 95% confidence intervals (CI) and numbers of exposed cases (ca) and controls (co) for risk factors for NHL patients only

**Factor**	**Category^a^**	**NHL patients univariate OR (95% CI) ca 33, co 66**	**NHL patients multivariate OR (95% CI)**
Decade of diagnosis (years)	1970–1979	1.00	1.00
		6, 10	
	1980–1989	0.65 (0.13–3.16)	0.86 (0.15–4.86)
		17, 32	
	1990–1999	0.16 (0.01–2.60)	0.22 (0.01, 4.04)
		10, 24	
			
Stage at diagnosis	Low	1.00	1.00
		17, 44	
	High	2.28 (0.97–5.36)	3.48 (0.91–13.26)
		16, 17	
			
Presenting site at diagnosis	Nodal disease	1.00	1.00
		20, 32	
	Extra-nodal disease	0.62 (0.26–1.43)	1.09 (0.34–3.53)
		13, 34	
	Other	—	—
		0, 0	
			
Treatment given	RT only	1.00	1.00
		13, 26	
	CT only	1.30 (0.50–3.37)	0.56 (0.15–2.16)
		12, 18	
	RT and CT	0.64 (0.19–2.18)	0.50 (0.11–2.29)
		5, 15	
	Surgery only/no treatment/treatment u/k	0.92 (0.20–4.14)	0.63 (0.10–3.99)
		3, 7	
Histology	High grade NHL	1.00	1.00
		16, 30	
	Other NHL	0.89 (0.39–2.04)	0.70 (0.25–1.98)
		17, 36	

a Reference category stated first.

—, odds ratio not presented as fewer than 5% of either cases or controls in the risk factor level.

**Table 4:** Odds ratios (OR), 95% confidence intervals (CI) and numbers of exposed cases (ca) and controls (co) for risk factors for HL patients only

**Factor**	**Category^a^**	**HL patients univariate OR (95% CI) ca 35, co 70**	**HL patients multivariate OR (95% CI)**
Decade of diagnosis (years)	1970–1979	1.00	1.00
		24, 40	
	1980–1989	0.45 (0.11–1.78)	0.31 (0.06, 1.56)
		11, 25	
	1990–1999	—	—
		0, 5	
			
Stage at diagnosis	Low	1.00	1.00
		26, 47	
	High	0.73 (0.31–1.73)	1.26 (0.33–4.74)
		9, 23	
			
Presenting site at diagnosis	Nodal disease	1.00	1.00
		34, 62	
	Extra-nodal disease	—	—
		0, 2	
	Other	—	—
		1, 6	
			
Treatment given	RT only	1.00	1.00
		18, 28	
	CT only	0.53 (0.18–1.55)	0.66 (0.13–3.28)
		7, 20	
	RT and CT	0.65 (0.22–1.95)	0.55 (0.16–1.89)
		9, 22	
	Surgery only/no treatment/treatment u/k	—	—
		1, 0	
			
Histology	Nodular sclerosing	1.00	1.00
		13, 20	
	Mixed cell	0.73 (0.29–1.87)	0.69 (0.25–1.88)
		14, 30	
	Other HL	0.63 (0.22–1.80)	0.65 (0.22–1.95)
		8, 20	
			
Splenectomy	No	1.00	1.00
		23, 51	
	Yes	1.48 (0.57–3.79)	1.51 (0.50–4.56)
		12, 19	

a Reference category stated first.

—, odds ratio not presented as fewer than 5% of either cases or controls in the risk factor level.
